# Alterations in amino acid metabolism during growth by *Staphylococcus aureus* following exposure to H_2_O_2_ – A multifactorial approach

**DOI:** 10.1016/j.heliyon.2018.e00620

**Published:** 2018-05-07

**Authors:** Grace R. Murphy, R. Hugh Dunstan, Margaret M. Macdonald, Johan Gottfries, Tim K. Roberts

**Affiliations:** a Metabolic Research Group, Faculty of Science and Information Technology, School of Environmental and Life Sciences, Department of Biology, University Drive, Callaghan, 2308, NSW, Australia; b Department of Chemistry, Gothenburg University, Sweden

**Keywords:** Microbiology, Metabolism

## Abstract

Temperature and pH are known to vary in a wound site due to the immune response and subsequent healing processes. This study used a multifactorial design to examine the cellular responses of *Staphylococcus aureus* to hydrogen peroxide (0–100 mM) when bacteria were grown in temperatures of 37 ± 2 °C and pH 7 ± 1, conditions potentially encountered in wound sites. A centroid sample was included in the design which represented the mid-point values of all three environmental parameters (37 °C, pH 7, 50 mM H_2_O_2_). Cytoplasmic extracts and corresponding medium supernatants were analysed for amino acid composition by gas chromatography. Exposures of *S. aureus* to H_2_O_2_ during the inoculation process resulted in extended lag phases lasting well after the peroxide had been neutralised by the bacterium's antioxidant systems, after which the bacteria eventually resumed growth at equivalent rates to the controls. Even though the subsequent growth rates appeared normal, the cells exhibited a variant metabolic regime at the mid-exponential phase of growth as a result of the initial exposure to peroxide. The alterations in metabolism were reflected by the differential amino acid profiles measured in the cytoplasmic extracts (P < 0.0001). The data indicated that the metabolic responses to H_2_O_2_ challenge were uniquely different depending on the variations of temperature and pH. The uptake patterns of amino acids from the media also altered depending on prevailing environmental conditions. From these results, it was proposed that a specific reproducible homeostasis could be induced under a specific set of defined environmental conditions. It was also evident that early toxic insults on the bacterial culture could have lasting impacts on cellular homeostasis after successive generations, even after the offending chemical had been removed and initial cell integrity restored. It was concluded that metabolic homeostasis would be continually adjusting and responding to changing environmental conditions to deploy defensive proteins as well as optimising processes for survival. The powerful ability to continually and rapidly adapt to the environment may represent the key feature supporting the virulence of *S. aureus* as an opportunistic pathogen invading the wound site.

## 1 Introduction

The genus *Staphylococcus* contains several species responsible for many nosocomial and community attained infections. The coagulase positive *Staphylococcus aureus* is renowned for causing significant infections in humans resulting in high morbidity and mortality. *S. aureus* can cause soft tissue infections and result in diseases such as osteomyelitis, meningitis, septic arthritis, food borne gastroenteritis, brain abscesses, cellulitis and septic shock (Archer, 1998; Clements and Foster, 1999). Between 25–35% of all cases of infective endocarditis are associated with *S. aureus* infection (Durack et al., 1994; Selton-Suty et al., 2012) as well as a high proportion of nosocomial infections from indwelling medical devices (Guerrero et al., 2009; Tong et al., 2015)

*S. aureus* has evolved with a range of virulence factors to support invasion, substrate utilisation and evasion of host defence systems. This includes an ability to exist within the host as an ongoing chronic infection, resisting host defence mechanisms, developing antibiotic resistance and residing as an intracellular infection in multiple tissue types (Proctor et al., 2015; von Eiff et al., 2006). *S. aureus* can be present as a commensal on human skin, but in the case of wounding or medical device insertion through the skin, the bacterium can take advantage of the physical break in the surface to invade tissues at the wound site (Foster, 2004).

*S. aureus* may be exposed to a range of variable environmental conditions when residing as a commensal bacterium on skin surfaces and more so when occupying wound sites. These environmental variations include alterations in pH, temperature, salt concentration and the presence of reactive oxygen species (ROS) such as H_2_O_2_. Low concentrations of H_2_O_2_ (100–250 μM) have been measured in the chronic wound site (Schäfer and Werner, 2008) whereas much higher concentrations can occur in a wound during the inflammatory response; it has been suggested that 115 nmoles of H_2_O_2_ were generated for every 10^6^ neutrophils during a respiratory burst (Nathan, 1987) although other studies suggest that this figure could be as high as 10 μmoles (Winn et al., 1991). Host temperature can range between 35 °C on the skin surface to 39 °C in localised, highly inflamed infections. The pH can alter significantly in wound sites (pH 5.4–8.9) as a result of the status of the healing process in the wound and it may also vary depending on the local tissue type (Romanelli, 1998; Tsukada et al., 1992; Wilson et al., 1978). The initial stages of healing generate a more basic pH (∼pH 8) in the repair zone; the pH becomes increasingly acidic (pH 5.4–5.6) as the tissues are repaired (Kaufman et al., 1985; Percival et al., 2014; Schneider et al., 2007). Infected wounds tend to have a higher pH with slower healing rates which suggests that alkaline conditions might be promoted by opportunistic bacterial pathogens (Schneider et al., 2007). Even though these conditions may not be ideal, they provide a continuing opportunity for bacterial colonisation.

The optimisation of metabolism under the varying environmental conditions found within a wound site would be critical to the invading pathogen attempting to undergo cell replication and penetration of host tissue while establishing an infection. There is little evidence in the literature on how *S. aureus* cells alter metabolism or structural components to optimise growth under the narrow ranges of pH, temperature, salt and oxidising bursts found in the wound site. A recent study on *S. lugdunensis* has shown that combinations of very limited parameter ranges led to substantial modifications in membrane composition and cell size (Crompton et al., 2014). Small variations in temperature (35–39 °C), pH (6–8) and NaCl (0–5%) resulted in pronounced changes in membrane fatty acid profiles. For example, the profile at 5% salt and pH 6 was very different to that observed at 5% salt and pH 8. When exposed to similar conditions, *S. aureus* showed both amino acid profile and proteome changes: increased NaCl (5%) at pH 6 resulted in a reduction in levels of many ribosomal proteins whilst 5% NaCl at pH 8 instead caused an upregulation in ribosomal proteins (Alreshidi et al., 2016). In a similar multifactorial study, fourier transform infrared (FTIR) spectroscopy aligned unique signature profiles for *S. aureus* grown under each different combination suggesting the development of unique phenotypes associated with each separate condition (Wehrli et al., 2014). These data support the hypothesis that staphylococcal species continually adapt to changing environments by altering membrane composition, structural components and cytoplasmic metabolism. To date this multifactorial design has yet to be adapted to investigate the changes in cytoplasmic metabolism that occur from reactive oxygen species released during immune responses.

The current study used a similar multifactorial experimental design (Alreshidi et al., 2016; Crompton et al., 2014; Wehrli et al., 2014) to explore the impact of alterations in the wound-site ranges of temperature (35–39 °C) and pH (6–8) on the metabolic responses of *S. aureus* to H_2_O_2_ exposure. The amino acids were selected as a critical pool of cytoplasmic metabolites since they are generally required to support synthesis of proteins, and specific amino acids are utilised for cell wall synthesis, nucleic acids and porphyrins, whereas others are readily used for energy substrates or precursors for osmoprotectants and other key metabolites. The aim was to test the hypothesis that varying conditions of temperature and pH would alter the metabolic homeostasis in the cytoplasm of *S. aureus* measured at the mid-exponential phase of growth, long after the ROS had been cleared following an early exposure to H_2_O_2_ at inoculation. The concentrations of cytoplasmic amino acids were measured to assess potential changes in amino acid homeostasis in the cell cytoplasm and growth media supernatants were assessed to investigate potential impacts on amino acid uptake characteristics.

## 2 Materials and methods

### 2.1 *S. aureus* strain maintenance and storage

American Type Culture Collection (ATCC) strain, ATCC® 29213™ was used for all experiments. ATCC® 29213™ strain was maintained on Columbia Horse Blood Agar (HBA) at 4 °C and stocks were preserved on sterile glass beads stored at −80 °C with subculturing as appropriate to maintain viability. Culture stocks were regularly tested for identity using bioMérieux API® *Staphylococcus* specific test kits.

### 2.2 Bacterial growth, cell harvesting and washing

*S. aureus* cells were grown in 50 mL of a synthetic defined medium (DM) in 250 mL conical flasks in an orbital shaking incubator at the prescribed temperatures of 35 °C, 37 °C and 39 °C as required. A defined medium was used instead of a complex commercial medium to enable GC-FID analyses of media supernatants to assess amino acid uptake and release following growth under test conditions. The defined medium contained amino acids, carbohydrates, vitamins and inorganic components in PBS as summarised in Table 1. The carbohydrate supplied was split across nine monosaccharides known to be utilised by *S. aureus* (Hengstenberg et al., 1969; Lally et al., 1985; Rosey et al., 1991) so that GC-FID analyses were not compromised by the presence of a single sugar that could overwhelm the analytical capacity of the column. Cells were harvested at the mid-exponential phase of growth by aseptically transferring each culture to a 50 mL capped polycarbonate centrifuge tube for centrifugation at 6,500 ×g at 4 °C for 15 minutes. To quantitate extracellular metabolites, the culture supernatants were filtered through a Millex® 0.22 μm membrane filter and stored at −20 °C until analysis. The harvested cells were resuspended in phosphate buffered saline (PBS), mixed and then centrifuged at 6,500 ×g at 4 °C for 15 minutes. This washing process was repeated twice more to ensure the removal of media residue. After washing, the cells were lyophilised to produce dry cell mass and weighed prior to extraction.

**Table 1:** The composition of the defined medium (DF) used in the experimental cultures to enable analyses of culture supernatants after growth under variable conditions to assess uptake and release of key amino acids.

Amino acids (mgL^−1^)		Carbohydrates (mgL^−1^)	Vitamins (mgL^−1^)	Inorganic salts (mgL^−1^)	Buffer components (gL^−1^)
L-Alanine **(**111) L-Asparagine (165) L-Aspartic acid (166) L-Arginine (174) L-Cystine (120) L-Glutamic acid (147) L-Glycine (150) L-Histidine (116) L-Isoleucine (164)	L-Lysine (183) L-Methionine (112) L-Phenylalanine (83) L-Proline (58) L-Serine (210) L-Threonine (238) L-Tryptophan (51) Tyrosine (45) Valine (105.44)	Fructose **(**160) Glucose (160) Mannose **(**160) Maltose **(**160) Ribose (160) Sucrose (160) Trehalose (160) Mannitol (160) Lactose **(**160)	Biotin **(**0.1) Nicotinic acid **(**2.3**)** D-pantothenic acid (2) Pyridoxal (2) Pyridoxamine 2HCl (2) Pyridoxine (8) Riboflavin (2) Thiamine HCl **(**2) Folic acid **(**0.01) p-aminobenzoic acid (0.2) Cyanocobalamine **(**0.1)	CaCl_2_5.0 (7) (NH_4_)_2_Fe(SO_4_)2.6H_2_O (7.05) MgSO_4_.7H_2_O (700) MnSO_4_.H_2_O **(**5.60)	NaCl (12) KCl (0.3) HNa_2_O_4_P (1.725) KH_2_PO_4_ (0.3)

### 2.3 Cold methanol water extraction

A protocol utilising cold methanol water was used for the extraction of cytoplasmic metabolites from harvested bacterial cells (Maharjan and Ferenci, 2003). The previously lyophilised and weighed cell mass (*c.* 10 mg) was suspended in 10 ml of 50% cold methanol water, vortexed and then frozen in liquid nitrogen. It was allowed to thaw in ice for 10 minutes and the contents centrifuged to remove cell debris. The supernatant was removed and placed in a clean 50 ml Falcon tube. The methanol extraction was repeated on the pellet residue and centrifuged before combining the second supernatant with the first supernatant. Tubes containing the supernatant extract were dried in a Labconco centrifugal vacuum concentrator to remove the methanol/water. The residue was derivatised appropriately for analysis by gas chromatography with flame ionisation detection (GC-FID).

### 2.4 Sample analysis with GC-FID

Amino acids from the extracellular culture supernatants and cytoplasmic extracts were extracted and processed with the EZ-faast sample kit for analyses by GC-FID. Norvaline (20 nmole) was added as an internal standard prior to each analysis. The column provided in the kit, a Zebron® 50% phenylpolysiloxane, 50% Methylpolysiloxane column (ZB-50), 10 m × 0.25 mm with 0.25 μm film thickness, was used to run all samples. A pulsed splitless method was used with a starting temperature of 150 °C which increased at 32 °C per minute to a holding temperature of 320 °C. The flow rate was two mL/minute and the carrier gas was Helium. The run time was 7.64 minutes in total. All GC-FID operations and quantifications were controlled through Agilent ChemStation^TM^ software with peak identification achieved by retention time comparisons with standards. Quantification was achieved using a four-point calibration table using the reference standard solutions provided by Phenomenex™.

### 2.5 Measuring hydrogen peroxide levels in liquid media

Quantofix® peroxide test sticks were used for the semi-quantitative determination of peroxide levels in solution. The sticks indicated levels of 0.0, 0.5, 2.5, 10 and >25 mg/L peroxide by dipping the strip into the solution for one second, waiting 15 seconds for the strip colour to change and comparing this to the colour chart provided. These test sticks were useful in determining if hydrogen peroxide remained in an experimental bacterial culture or if it had been completely degraded.

In order to gain sufficient bacterial mass for extraction and analysis, densities of bacterial cells exceeding that which would be expected in a wound site were required. Thus, in this initial investigation, the impact of exposure to a range of peroxide concentrations was undertaken to determine an appropriate dose range at the required cell densities that would lead to observable impacts on the growth rate of *S. aureus*. Assessment of the minimal inhibitory concentrations (MIC) of hydrogen peroxide was not feasible as *S. aureus* has effective anti-oxidant systems (Cosgrove et al., 2007; Mandell, 1975) which readily removed increasing concentrations of H_2_O_2_ from culture media and allowed for the resumption of growth. To determine a useful dose response curve, concentrations of H_2_O_2_ were added to the defined medium to give final concentrations ranging in the first experiment from 0 to 100 μM and in the second experiment, from 0 to 90 mM (in triplicate). Aliquots of 190 μL were then added to a 96 well plate which was inoculated with 10 μl of bacterial suspension from an overnight broth and incubated at 37 °C. The absorbance of each well was measured at time 0 and at 30 minute intervals for a total of 180 minutes.

### 2.6 Multifactorial experiment

**Fig. 1:**
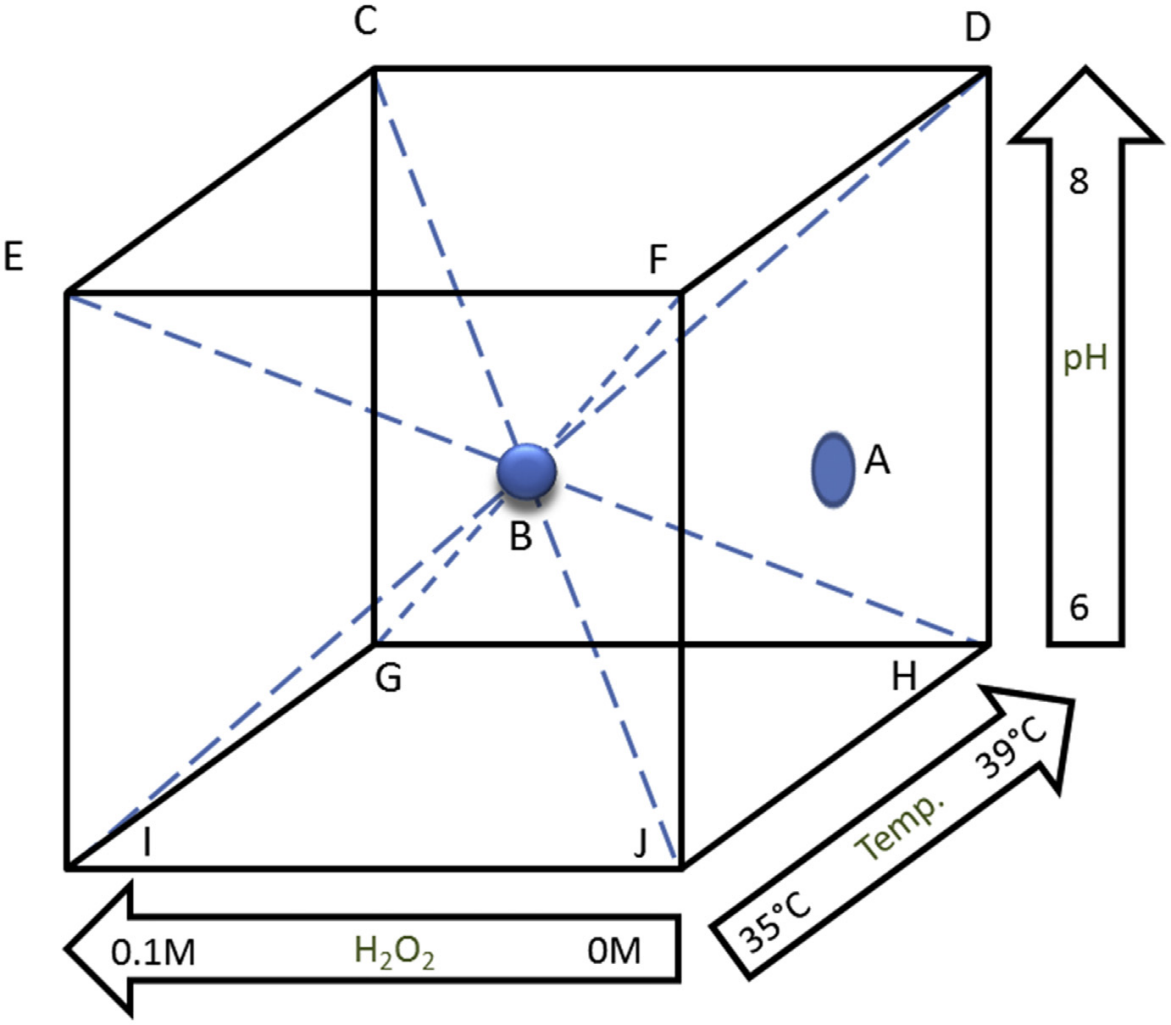
The cuboid experiment design whereby each corner of the cube represents a specific variable combination that was tested. The centre sphere (B) represents the centroid experiment where all tested variables directly intersect. The reference control (A) exhibits optimal conditions for *S. aureus* growth. The other experimental parameters are as follows: 39 °C, pH 8 and 100 mM H_2_O_2_ (C), 39 °C, pH 8 and 0 mM H_2_O_2_ (D), 35 °C, pH 8 and 100 mM H_2_O_2_ (E), 35 °C, pH 8 and 0 mM H_2_O_2_ (F), 39 °C, pH 6 and 100 mM H_2_O_2_ (G), 39 °C, pH 6 and 0 mM H_2_O_2_ (H), 35 °C, pH 6 and 100 mM H_2_O_2_ (I), 35 °C, pH 6 and 0 mM H_2_O_2_ (J).

An experiment was designed to examine the potential impacts on metabolism of *S. aureus* in response to changes in combinations of three environmental parameters. These variables included temperature (35–39 °C), pH (6–8) and hydrogen peroxide concentrations (0–100 mM) shown to extend the lag phase of growth of *S. aureus*. The experiment aimed to collect data from eight different variable combinations, as represented by Fig. 1, for comparison with a set of optimal conditions representing a reference control combination (A) which was carried out at the beginning (n = 4), midpoint (n = 4) and end (n = 4) of the experimental period generating a total of 12 replicates. This allowed for temporal variations based on timing logistics. In addition to these nine data points, a tenth sample series was assessed which represented the midpoint of all experimental ranges of the environmental parameters (37 °C, pH 7 and 50 mM H_2_O_2_). This set of samples was referred to as the centroid (Fig. 1, the centre of the cube designated B) and was repeatedly assessed throughout the experimental period with the reference control to generate 12 replicates. Data points C and D were completed in triplicate and each of the remaining data points (E–J) within this multifactorial design were completed in quadruplicate.

The multifactorial experiment was carried out over 35 days and included 14 separate experiments that represented the sets of conditions in the eight “corners” of the cube combined with three separate control and centroid experiments. The control and centroid experiments were repeated on day 1, day 15 and day 35 to control for variations that might occur throughout the whole experimental period. Each of the separate experiments was carried out in quadruplicate to optimise statistical evaluation.

For each experiment of the multifactorial design, 2.5 mL from an overnight starter culture were added to 47.5 mL of defined medium. The pH and H_2_O_2_ concentration were then altered appropriately and the flasks placed on an orbital shaking incubator (120 rpm) which was set to temperatures of 35 °C, 37 °C or 39 °C. Absorbance (A_600_) measurements were taken aseptically at hourly intervals until the bacterial cells had reached mid-exponential phase, after which the cells were harvested, washed and lyophilised. Supernatant samples were filtered through a 0.22 μm membrane syringe filter and frozen until analysed by GC-FID.

### 2.7 Statistical analyses

Dell Statistica (data analysis software system), version 13, software.dell.com, Dell Inc. (2015) was used for all analyses of variance (ANOVA) to determine statistical significance and for the generation of 2D and 3D canonical plots for discriminant function analysis of the multifactorial experiment data. Levels of statistical significance were set at *p* < 0.05. Umetric's Modde™ 10.1 was used for the generation of 4D response contour plots to show the concentrations of intracellular and excreted metabolites produced under different environmental conditions.

## 3 Results

An initial experiment performed to test the impact of H_2_O_2_ concentrations in the range reported for wound sites (10–100 μM) on bacterial growth at the high cell densities in 200 μL cultures in a 96 well plate found no measurable impact on bacterial growth. Since the cell density of the inoculum would be far higher than the numbers of cells present invading the wound site, it was likely that the 10–100 μM was too low to elicit an effect on culture growth. It was thus necessary to determine the capacity of the higher cell densities of the *S. aureus* culture inoculum to remove H_2_O_2_ from the defined media *via* intrinsic antioxidant protection systems.

A 5% inoculum from an overnight starter culture was used to freshly inoculate a 50 mL culture of *S. aureus* which was incubated at 37 °C in defined medium (DM) in the presence of 50 mM H_2_O_2._ Assessments by the Quantofix® peroxide test sticks at eight minute intervals revealed that the culture was completely free of peroxide by 16 minutes. Peroxide was still present after five days in the control sample which consisted of defined medium loaded with 50 mM H_2_O_2_ inoculated with sterile PBS. Given the rapid removal of 50 mM H_2_O_2_ from the defined medium by the bacterial inoculum, triplicate cultures in DM containing 50 mM H_2_O_2_ were incubated and assessed for potential impact on the growth characteristics in comparison with control cultures with no H_2_O_2_. The control cultures had a lag phase of approximately one hour and an exponential phase generation time of 79 minutes (±0.54) in the defined medium whereas the cultures inoculated with H_2_O_2_ had an extended lag phase of approximately two hours with an eventual mean exponential phase generation time of 81 minutes (±0.46). The lag phase of the H_2_O_2_ treated cultures extended well beyond the 16 minute period of hydrogen peroxide depletion as well as the lag phase of the control; there was no significant difference between the exponential phase generation times between the treatment and control.

**Fig. 2:**
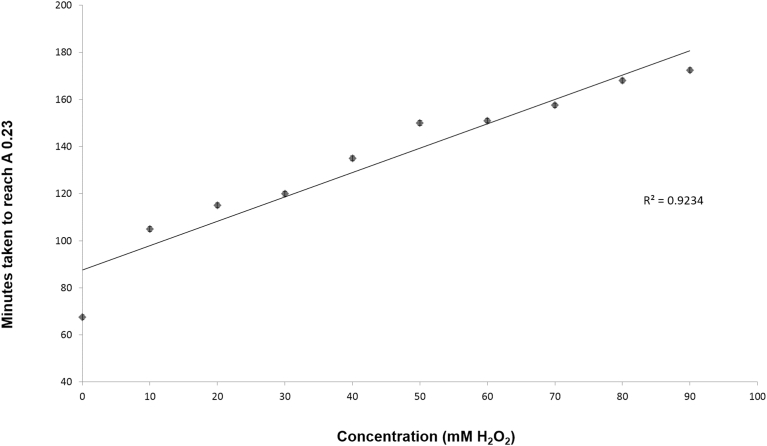
Dose response curve for the action of H_2_O_2_ on delaying the growth of *S. aureus* to reach a target absorbance (A_600_) of 0.23, representing the early exponential phase of growth (n = 3, mean ± SE).

On the basis of these results, the assessment of the minimal inhibitory concentration (MIC) for H_2_O_2_ required a non-standard approach; whilst present in the medium, H_2_O_2_ slowed or inhibited bacterial growth but, once cleared *via* anti-oxidant systems, cell cultures showed an extended lag phase before resuming normal growth rates. The approach taken was thus to assess a dose response curve in terms of the impact of the H_2_O_2_ concentrations on the time taken to reach an absorbance value representative of the early exponential phase of growth. A 96 well plate was used to inoculate cultures of *S. aureus* which were exposed to concentrations of H_2_O_2_ ranging from 0‒90 mM. The wells were filled with the 190 μL DM containing final concentrations of 0, 10, 20, 30, 40, 50, 60, 70, 80 and 90 mM H_2_O_2_ and inoculated with a 10 μl inoculum from a starter culture. The well cultures were incubated at 37 °C and absorbance measured at time zero and at 30 minute intervals to assess the times taken to reach the value of A_600_ = 0.23 or early exponential growth phase. The results summarised in Fig. 2 demonstrated a linear relationship between increasing H_2_O_2_ concentrations and increased times required to reach the target stage of growth.

**Fig. 3:**
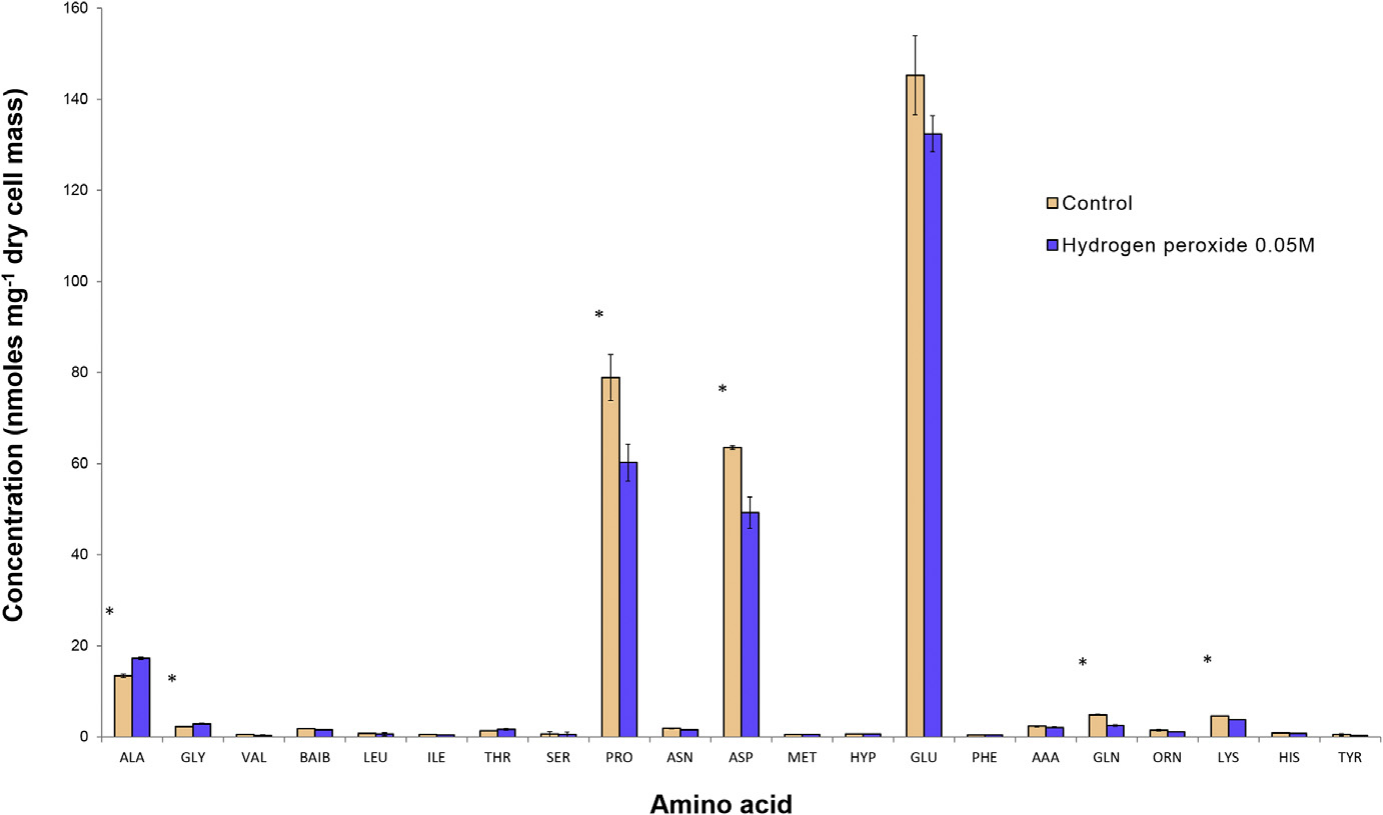
Comparison of the cytoplasmic amino acids of *S. aureus* cells grown under optimal control conditions or in the presence of 50 mM H_2_O_2_ which were harvested at the mid-exponential phase of growth (n = 3, mean ± SE). * = P < 0.05.

Control cells grown on DM and those exposed to 50 mM H_2_O_2_ at inoculation were incubated and harvested at the mid-exponential phase for the extraction and analyses of cytoplasmic amino acids. The amino acid profiles summarised in Fig. 3 showed that the cytoplasm from both control cells and those exposed to H_2_O_2_ had glutamic acid, proline, aspartic acid and alanine as the major cytoplasmic amino acid components. Comparisons between the control and H_2_O_2_ treatments revealed that the concentrations of proline and aspartic acid as well as the lower abundance components glutamine and lysine were significantly decreased in the H_2_O_2_ treatment group (P < 0.05). Conversely, alanine and glycine increased significantly in the H_2_O_2_ treated cultures (P < 0.05). Analyses of media supernatant collected at the mid-exponential phase of growth revealed that cells exposed to H_2_O_2_ had a significantly lower level of total amino acids remaining in the medium at 9.13 mM compared with 11.63 mM remaining in the control medium (P < 0.05), with specific reductions in the levels noted for alanine, glycine, leucine, isoleucine, proline, methionine, lysine, tyrosine and cystine.

A multifactorial experiment was designed to investigate the potential influences of varying temperature and pH on the response by cells to the initial exposures of H_2_O_2_ during growth. The experimental cultures were set up under temperature and pH conditions consistent with those reported for the human wound site where temperatures ranged from 35 °C to 39 °C and pH from 6 to 8. The H_2_O_2_ concentrations used were higher than those reported in the wound site to cater for the higher cell densities required in this experiment to enable analyses of cytoplasmic extracts (Fig. 1). The control set of samples (A) represented growth under optimal conditions of 37 °C, pH 7, and 0 mM H_2_O_2_ and the centroid set of samples (B) represented the midpoint of all experimental ranges, differing from the control (A) only by the addition of 50 mM H_2_O_2_ (37 °C, pH 7, 50 mM H_2_O_2_). After reaching mid-exponential phase, cytoplasmic extracts from each sample were analysed to determine the amino acid profiles for each of the experimental groups.

**Fig. 4:**
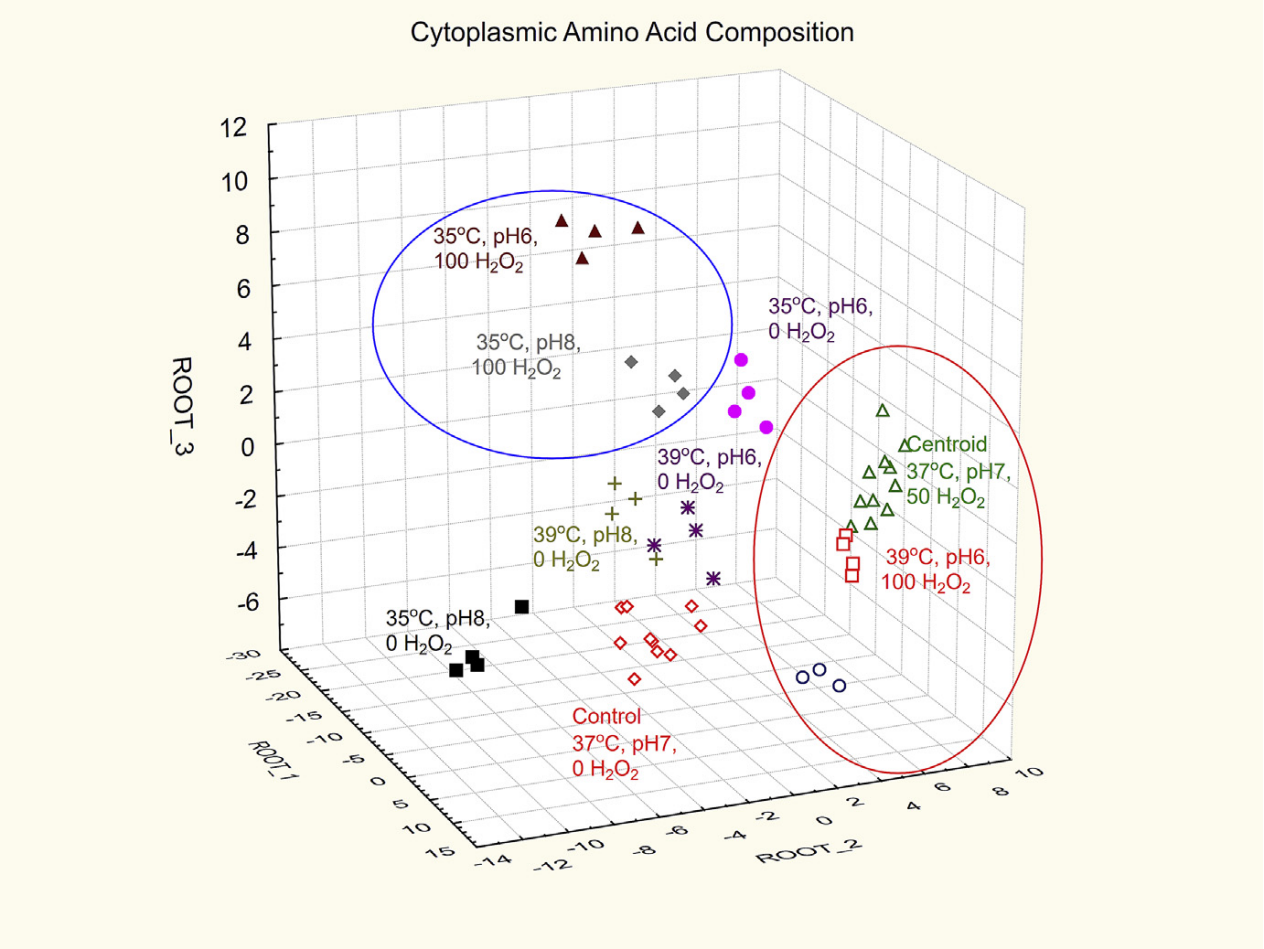
3D canonical plot from the discriminant function analyses applied to the cytoplasmic amino acid profiles from each of the 10 experimental groups. Each case replicate has been colour coded and labelled for the experiments to show grouping.

Multivariate analyses using standard discriminant function revealed that there were significant differences between the amino acid composition profiles from the different treatment groups (Wilk's Lambda < 0.0001, F remove = 13.954, P < 0.0001). Each set of cells derived from the different growth regimes generated a characteristic cytoplasmic amino acid profile. The amino acid compositions from each sample were processed via the discriminant function to generate a score (root 1) that generates maximal separation between the groups of samples. The next most effective function for discrimination between the samples in an orthogonal direction was used to generate the root 2 values, and a third function to generate root 3 values. These root scores were then used to position each sample onto a 3-D canonical plot as shown in Fig. 4. The use of colour coding each group of sample allowed visualisation of the tight clustering of replicate samples within each group which showed complete resolution between treatment sets. The control and centroid replicates were analysed on three separate occasions at the beginning, middle and end of the experimental period. The tight clustering of these sets of 12 samples in each of the control and centroid sets illustrates the reproducibility of the cytoplasmic amino acid profiles generated under defined sets of growth conditions on different days. The cells exposed to 100 mM H_2_O_2_ at 35 °C with pH 6 and pH 8 have been circled in blue in Fig. 4 to show that they are well separated from the equivalent cultures at 39 °C and the centroid sample which had 50 mM H_2_O_2_ at 37 °C and pH 7 (circled in red). The control and 0 mM H_2_O_2_ treatments at the various temperature and pH regimes were all separated from each other but were presented as a central core between the two 100 mM treatment regimens at 35 °C and 39 °C. The cultures grown at 35 °C without exposure to H_2_O_2_ at pH 6 displayed very strong differences between the equivalent cells grown at pH 8.

**Fig. 5:**
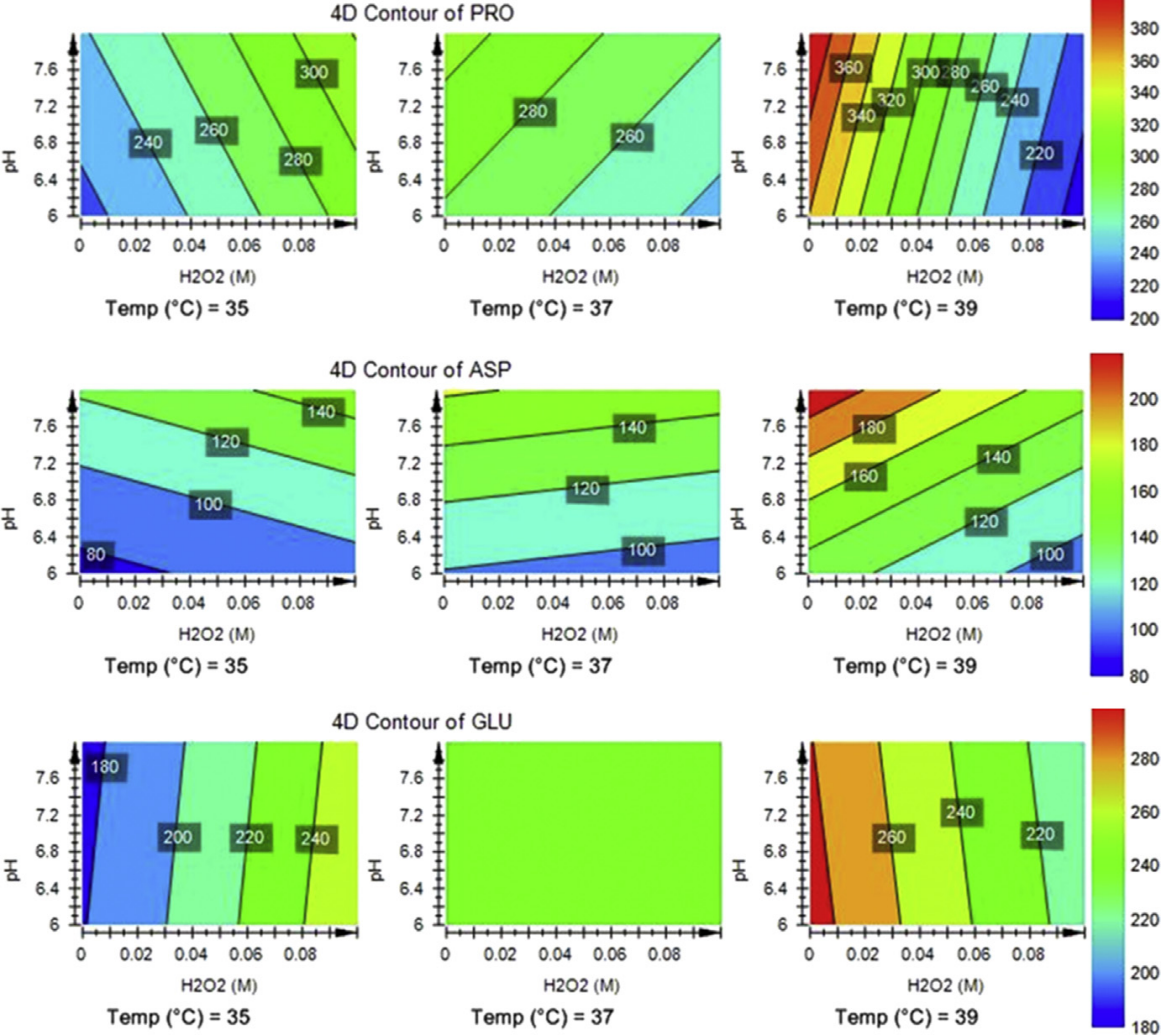
4D response contour plots which show the extrapolated impact on cytoplasmic concentrations of proline (PRO top), aspartic acid (ASP middle) and glutamic acid (GLU bottom) by simultaneously adjusting pH and H_2_O_2_ at 35 °C, 37 °C and 39 °C. The scale for concentration is given in nmoles mg^−1^ of dried cell mass.

The design of the experiments also allowed an investigation into a fourth dimensional component of analyses where the concentrations of any given metabolite can be assessed against the scalar contributions of two variables across three different temperature ranges. This was achieved by plotting the data in the contour plots shown in Fig. 5. Proline, aspartic acid and glutamic acid were the most abundant amino acids in the cytoplasm collectively comprising 93% of the amino acid pool under control conditions. The highest abundance of proline was observed in the cytoplasm from cells grown at 39 °C, whereas cells grown at 35 °C and 37 °C displayed low to mid-range concentrations (Fig. 5). At 35 °C, increases in H_2_O_2_ concentration led to increased proline levels with some influence from changes in pH. At 37 °C, the opposite effect was observed where higher concentrations of H_2_O_2_ led to decreases in intracellular proline. At 39 °C, the level of proline was highest at 0 mM H_2_O_2_ and increasing concentrations of H_2_O_2_ led to marked reductions in cytoplasmic levels.

Aspartic acid displayed a progressive change in homeostatic characteristics between the cells at 35 °C and those at 39 °C. At 35 °C, increasing levels of aspartic acid were weakly associated with increasing pH and to a lesser extent with H_2_O_2_ concentration. At 37 °C, changes in pH primarily altered cytoplasmic levels of aspartic acid and H_2_O_2_ had little or no effect. At 39 °C, increasing pH resulted in the highest levels of aspartic acid whilst increasing H_2_O_2_ was correlated with decreasing cytoplasmic aspartic acid. At 35 °C, increasing levels of glutamic acid were associated with increasing levels of H_2_O_2_ but pH showed little to no effect. At 37 °C, neither H_2_O_2_ nor pH influenced the cytoplasmic concentrations of glutamic acid. At 39 °C, increasing H_2_O_2_ was associated with a reduction in levels of glutamic acid with pH showing little to no effect.

**Fig. 6:**
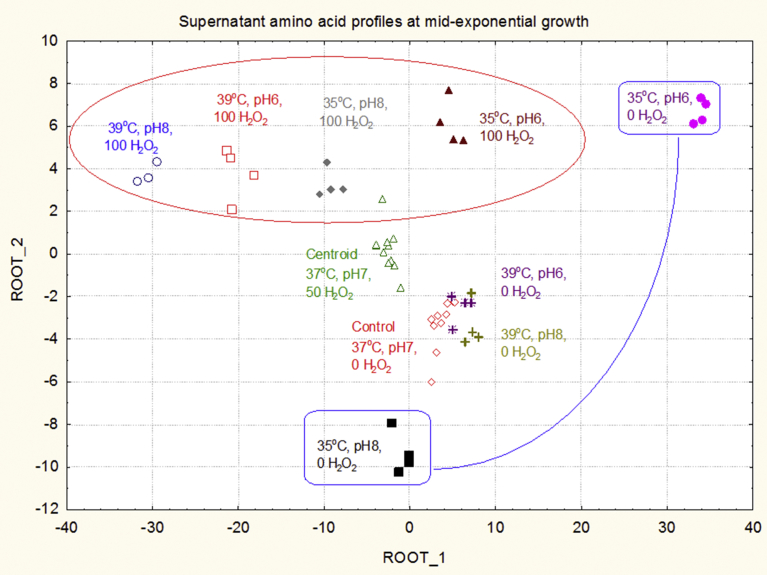
2D canonical plot from the discriminant function analyses applied to the supernatant amino acid profiles from each of the 10 experimental groups. Each case replicate has been colour coded and labelled for the experiments to show grouping. The contrast between the amino acid uptake profiles for cells grown at either pH 6 or pH 8 at 35 °C without peroxide has been highlighted.

Equivalent multivariate analyses were performed on the corresponding amino acid composition profiles of culture supernatants to explore alterations in external amino acid levels in the medium. The canonical plot of the root 1 and root 2 scores for each medium supernatant sample was generated in Fig. 6. This plot shows that the supernatant amino acid profiles for the control were similar and were in close proximity with those derived from the cells grown with no H_2_O_2_ at 39 °C at either pH 6 or pH 8. This suggested that at 39 °C in the absence of H_2_O_2_, the amino acid uptake patterns were only minimally affected by changes in pH in comparison with the control. A decrease of 4 °C to 35 °C with no H_2_O_2_ at pH 6 and pH 8 however, resulted in significant changes in patterns of amino acid utilisation, as highlighted in blue in Fig. 6, which was consistent with the equivalent alterations in cytoplasmic composition between these cultures shown in Fig. 4. The cells exposed to 100 mM H_2_O_2_ have been circled in red in Fig. 6 to show that they are well separated from the control, centroid and 0 mM H_2_O_2_ treatments. Within this broad grouping, each of the temperature and pH-mediated responses to the 100 mM H_2_O_2_ treatments were well separated by their cytoplasmic amino acid profiles. It was interesting to note that the centroid set of conditions which had 50 mM H_2_O_2_ was positioned between the 100 mM H_2_O_2_ treatments and the control samples. These results indicated that growth under varying conditions altered the uptake patterns of amino acids into the culture media with significant differences recorded between groups for the major cytoplasmic components including proline, glutamic acid/glutamine, aspartic acid, alanine and glycine.

## 4 Discussion

The results of this study clearly demonstrated that *S. aureus* had different metabolic responses to exposures of H_2_O_2_ depending on variations in the environmental temperature and pH conditions. The cytoplasmic amino acids represent a key pool of metabolites since they are utilised in endogenous respiration as well as providing vital precursors for the synthesis of complex macromolecules such as proteins, nucleotides, phospholipids and cell wall components (Mah et al., 1967). Cytoplasmic amino acid profiles represented biochemical snapshots of metabolic homeostasis and comparisons of cytoplasmic extracts at the mid-exponential growth phase revealed significant reductions in two of the major components, proline and aspartic acid (Fig. 3) as well as in glutamine and lysine and concomitant increases in alanine and glycine. The early exposure of cells to peroxide during inoculation thus resulted in changes in cytoplasmic amino acid composition at a time well after hydrogen peroxide was cleared from the culture media. This suggested that exposure to H_2_O_2_ led to long-standing changes in metabolic homeostasis, well after the challenge had been removed. These changes were reflected in lower intracellular levels of specific amino acids which may have been utilised at higher rates to support protein synthesis for the direct repair of damaged proteins following exposure to H_2_O_2_. The lower amino acid levels may also represent an upregulation of certain proteins to support the synthesis of anti-oxidant systems and to facilitate the repair of damaged cellular components such as nucleic acids and membranes (Pacifici and Davies, 1990; Wolf et al., 2008). This interpretation whereby the cells evidently require a program of repair and recovery prior to restarting growth, was consistent with the observation that the bacteria exposed to H_2_O_2_ had utilised more of the amino acids available in the medium compared with the cells grown without H_2_O_2_.

The multivariate analyses of the data indicated that small variations in temperature and pH could differentially alter the profiles of amino acids in the cytoplasm of *S. aureus* following exposures to H_2_O_2_ (Fig. 4)*.* It was concluded that *S. aureus* was very sensitive to fluctuations in environmental conditions and implemented the most advantageous metabolic response mechanisms for optimal survival in a dynamic environment. The specific metabolic responses to H_2_O_2_ under one set of environmental conditions differed from responses under other sets of conditions within the temperature (37 ± 2 °C) and pH (7 ± 1) ranges. Under similarly restricted temperature and pH conditions, *S. aureus* and *S. lugdunensis* have also been found to respond differentially to osmotic challenge with resultant alterations in protein and membrane composition and cell morphology (Alreshidi et al., 2016; Crompton et al., 2014; Wehrli et al., 2014). In the current study, examples of the differential responses to H_2_O_2_ under various temperature and pH conditions were demonstrated for the major cytoplasmic components glutamic acid, proline and aspartic acid (Fig. 5). Not only were the responses altered within each change of temperature and pH but the amino acids showed differential response characteristics compared to each other, indicating that their cytoplasmic levels were under independent control with respect to cytoplasmic concentration. Furthermore, the patterns of amino acid utilisation or uptake from the media were different among each variation in temperature, pH and H_2_O_2_ exposure (Fig. 6). These data provided evidence that specific nutrient requirements were utilised to support the discrete repair mechanisms and establish the optimal metabolic homeostasis required to cope with defined sets of environmental conditions.

In the context of the wound site, H_2_O_2_ can be released by neutrophils during a respiratory burst (Nathan, 1987) where concentrations of H_2_O_2_ from 100‒250 μM have been measured (Schäfer and Werner, 2008). In the present study, this level of H_2_O_2_ was not sufficient to elicit an inhibitory effect due to the higher densities of cells presented under the broth culture conditions required for effective analysis of the cytoplasmic amino acids. The innate anti-oxidant protection systems within staphylococci including superoxide dismutases, catalases and pigments (Wolf et al., 2008), were apparently very effective in the removal of the 0–100 μM H_2_O_2_ when presented to the higher cell densities. On this basis, inoculation of *S. aureus* into a culture medium containing H_2_O_2_ in the 10–90 mM range was investigated and caused a concentration-dependent increase in the lag phase compared with that observed in control cultures grown without H_2_O_2_. The delay extended well beyond the 16 minutes required to remove all trace of the 50 mM H_2_O_2_ added to the culture, with the lag phase effectively doubling from approximately one hour in the control to two hours in the cultures exposed to peroxide. This delay and subsequent resumption of growth in *S. aureus* has been previously reported in response to sub-lethal (25, 30 mM) levels of H_2_O_2_ (Painter et al., 2015). It was proposed that the addition of this level of H_2_O_2_ caused oxidative damage to key components such as proteins, nucleic acids and cell membranes as described previously (Hassett and Cohen, 1989; Linley et al., 2012) and the cells thus required an extended lag period to facilitate repair and adjustment to the new conditions. This lengthening of the lag phase due to H_2_O_2_ would likely involve the induction of an SOS response in *S. aureus* initiating DNA repair (Anderson et al., 2006; Žgur-Bertok, 2013). Genes that contribute to fermentative metabolism have also been shown to be induced following exposure to H_2_O_2_ which suggests that *S. aureus* may enter into a partially anaerobic or oxygen limiting state to minimize oxidative damage during repair (Chang et al., 2006).

The results from the dose response analysis indicated that the higher the level of H_2_O_2_ supplied, the longer it took for cell cultures to reach the designated target stage of growth. It has also previously been shown that prolonged exposure to sub-lethal levels of H_2_O_2_ (25, 30 mM) and the subsequent SOS response in *S. aureus* leads to an increase in a SCV subpopulation with SCV cells showing acquired resistance to H_2_O_2_ through enhanced catalase production (Painter et al., 2015). In the present study, following an elongated lag phase the cells treated with H_2_O_2_ attained growth rates that were similar to the control. Although the subsequent growth rates appeared normal, the cells exhibited a variant metabolic regime as a result of the initial exposure to peroxide, which was reflected in the altered amino acid composition profiles measured in the cytoplasmic extracts (Fig. 3). At lower population densities, such as those found in the wound site, the μM H_2_O_2_ range may be effective in limiting bacterial growth. In addition, the concentrations of H_2_O_2_ may be high in localised micro-environments around or near the respiratory burst. During this period of bacteriostasis, bacterial virulence may be impaired for a sufficient time to enable the host to further defend against infection.

From these results, it was proposed that a specific reproducible homeostasis could be induced under any defined set of environmental conditions. The corollary of this observation is that homeostasis would be continually adjusting and responding to changing environmental conditions; this may involve regulation of gene expression to deploy defensive proteins as well as optimising regulation of existing systems by feedback inhibition and activation of current proteins. The powerful ability to continually and rapidly adapt to the environment may represent the key survival feature supporting the virulence of *S. aureus* as an opportunistic pathogen invading the wound site.

## Declarations

### Author contribution statement

R. Hugh Dunstan: Conceived and designed the experiments; Analyzed and interpreted the data; Contributed reagents, materials, analysis tools or data; Wrote the paper.

Margaret M. Macdonald: Conceived and designed the experiments; Contributed reagents, materials, analysis tools or data; Wrote the paper.

Johan Gottfries: Analyzed and interpreted the data; Contributed reagents, materials, analysis tools or data; Wrote the paper.

Tim K. Roberts: Conceived and designed the experiments; Wrote the paper.

### Funding statement

The work was supported by the Gideon Lang Research Foundation. The funders had no role in study design, data collection and analysis, decision to publish, or preparation of the manuscript.

### Competing interest statement

The authors declare no conflict of interest.

### Additional information

No additional information is available for this paper.

## References

[bib1] Alreshidi M.M. (2016). Changes in the cytoplasmic composition of amino acids and proteins observed in *Staphylococcus aureus* during growth under variable growth conditions representative of the human wound site. PLoS One.

[bib2] Anderson K.L. (2006). Characterization of the *Staphylococcus aureus* heat shock, cold shock, stringent, and SOS responses and their effects on log-phase mRNA turnover. J. Bacteriol..

[bib3] Archer G.L. (1998). *Staphylococcus aureus*: a well-armed pathogen. Clin. Infect. Dis..

[bib4] Chang W., Small D.A., Toghrol F., Bentley W.E. (2006). Global transcriptome analysis of *Staphylococcus aureus* response to hydrogen peroxide. J. Bacteriol..

[bib5] Clements M.O., Foster S.J. (1999). Stress resistance in *Staphylococcus aureus*. Trends Microbiol..

[bib6] Cosgrove K., Coutts G., Jonsson M., Tarkowski A., Kokai-Kun J.F., Mond J.J., Foster S.J. (2007). Catalase (KatA) and alkyl hydroperoxide reductase (AhpC) have compensatory roles in peroxide stress resistance and are required for survival, persistence, and nasal colonization *in Staphylococcus aureus*. J. Bacteriol..

[bib7] Crompton M.J., Dunstan R.H., Macdonald M.M., Gottfries J., von Eiff C., Roberts T.K. (2014). Small changes in environmental parameters lead to alterations in antibiotic resistance, cell morphology and membrane fatty acid composition in *Staphylococcus lugdunensis*. PLoS One.

[bib8] Durack D.T., Lukes A.S., Bright D.K., Service D.E. (1994). New criteria for diagnosis of infective endocarditis: utilization of specific echocardiographic findings. Am. J. Med..

[bib9] Foster T.J. (2004). The *Staphylococcus aureus* “superbug”. J. Clin. Invest..

[bib10] Guerrero M.L.F., López J.J.G., Goyenechea A., Fraile J., de Górgolas M. (2009). Endocarditis caused by *Staphylococcus aureus*: a reappraisal of the epidemiologic, clinical, and pathologic manifestations with analysis of factors determining outcome. Medicine.

[bib11] Hassett D.J., Cohen M.S. (1989). Bacterial adaptation to oxidative stress: implications for pathogenesis and interaction with phagocytic cells. Faseb. J..

[bib12] Hengstenberg W., Penberthy W., Hill K.L., Morse M. (1969). Phosphotransferase system of *Staphylococcus aureus*: its requirement for the accumulation and metabolism of galactosides. J. Bacteriol..

[bib13] Kaufman T., Eichenlaub E., Angel M., Levin M., Futrell J. (1985). Topical acidification promotes healing of experimental deep partial thickness skin burns: a randomized double-blind preliminary study. Burns.

[bib14] Lally R.T., Ederer M., Woolfrey B. (1985). Evaluation of mannitol salt agar with oxacillin as a screening medium for methicillin-resistant *Staphylococcus aureus*. J. Clin. Microbiol..

[bib15] Linley E., Denyer S.P., McDonnell G., Simons C., Maillard J.-Y. (2012). Use of hydrogen peroxide as a biocide: new consideration of its mechanisms of biocidal action. J. Antimicrob. Chemother..

[bib16] Mah R.A., Fung D.Y., Morse S.A. (1967). Nutritional requirements of *Staphylococcus aureus* S-6. J. Appl. Microbiol..

[bib17] Maharjan R., Ferenci T. (2003). Global metabolite analysis: the influence of extraction methodology on metabolome profiles of *Escherichia coli*. Anal. Biochem..

[bib18] Mandell G. (1975). Catalase, superoxide dismutase, and virulence of *Staphylococcus aureus*. In vitro and in vivo studies with emphasis on staphylococcal–leukocyte interaction. J. Clin. Invest..

[bib19] Nathan C.F. (1987). Neutrophil activation on biological surfaces. Massive secretion of hydrogen peroxide in response to products of macrophages and lymphocytes. J. Clin. Invest..

[bib20] Pacifici R.E., Davies K.J.A., Lester Packer A.N.G. (1990). Protein degradation as an index of oxidative stress.

[bib21] Painter K.L., Strange E., Parkhill J., Bamford K.B., Armstrong-James D., Edwards A.M. (2015). *Staphylococcus aureus* adapts to oxidative stress by producing H_2_O_2_-resistant small-colony variants via the SOS response. Infect. Immun..

[bib22] Percival S.L., McCarty S., Hunt J.A., Woods E.J. (2014). The effects of pH on wound healing, biofilms, and antimicrobial efficacy. Wound Repair Regen..

[bib23] Proctor R.A., Kriegeskorte A., Kahl B.C., Becker K., Löffler B., Peters G. (2015). *Staphylococcus aureus* Small Colony Variants (SCVs): a road map for the metabolic pathways involved in persistent infections Host-adapted metabolism and its regulation in bacterial pathogens. Cell Infect. Microbiol..

[bib24] Romanelli M. (1998). Evaluation of surface pH on venous leg ulcers under Allevyn dressings. International Congress and Symposium Series-royal Society of Medicine, 1998.

[bib25] Rosey E., Oskouian B., Stewart G. (1991). Lactose metabolism by *Staphylococcus aureus*: characterization of lacABCD, the structural genes of the tagatose 6-phosphate pathway. J. Bacteriol..

[bib26] Schäfer M., Werner S. (2008). Oxidative stress in normal and impaired wound repair. Pharmacol. Res..

[bib27] Schneider L.A., Korber A., Grabbe S., Dissemond J. (2007). Influence of pH on wound-healing: a new perspective for wound-therapy?. Arch. Dermatol. Res..

[bib28] Selton-Suty C. (2012). Preeminence of *Staphylococcus aureus* in infective endocarditis: a 1-year population-based survey. Clin. Infect. Dis..

[bib29] Tong S.Y., Davis J.S., Eichenberger E., Holland T.L., Fowler V.G. (2015). *Staphylococcus aureus* infections: epidemiology, pathophysiology, clinical manifestations, and management. Clin. Microbiol. Rev..

[bib30] Tsukada K., Tokunaga K., Iwama T., Mishima Y. (1992). The pH changes of pressure ulcers related to the healing process of wounds. Wounds.

[bib31] von Eiff C., Peters G., Becker K. (2006). The small colony variant (SCV) concept—the role of staphylococcal SCVs in persistent infections. Injury.

[bib32] Wehrli P.M., Lindberg E., Angerer T.B., Wold A.E., Gottfries J., Fletcher J.S. (2014). Maximising the potential for bacterial phenotyping using time-of-flight secondary ion mass spectrometry with multivariate analysis and Tandem mass spectrometry. Surf. Interface Anal..

[bib33] Wilson I., Henry M., Quill R., Byrne P. (1978). The pH of varicose ulcer surfaces and its relationship to healing. J. Vasc. Dis..

[bib34] Winn J.S., Guille J., Gebicki J.M., Day R.O. (1991). Hydrogen peroxide modulation of the respiratory burst of human neutrophils. Biochem. Pharmacol..

[bib35] Wolf C., Hochgräfe F., Kusch H., Albrecht D., Hecker M., Engelmann S. (2008). Proteomic analysis of antioxidant strategies of *Staphylococcus aureus*: diverse responses to different oxidants. Proteomics.

[bib36] Žgur-Bertok D. (2013). DNA damage repair and bacterial pathogens. PLoS Pathog..

